# Supplementary material for: Fish Protein Hydrolysate as Protein Enrichment in Texture-Modified Salmon Products
Source: Foods. 2025 Jan 8;14(2):162. doi: 10.3390/foods14020162 (PMC11764784; doi:10.3390/foods14020162)
Supplement: Supplementary file 1 [file foods-14-00162-s001.zip › foods-3339335-supplementary.pdf]

Supplementary material

Table S1

Attributes and definition used in the sensory evaluation (QDA®) assessed in intensity scale 1 (low intensity) to 9 (high intensity).

Attribute	Definition
Odour	
Sour odour	Relates to a fresh, balanced odour due to the presence of organic acids.
Sweet odour	Relates to the basic taste sweet (sucrose)
Metallic odour	Relates to odour of metal (ferrous sulphate)
Dairy odour	Relates to odour of dairy products like cream and sour cream.
Spice odour	Relates to odour of spices like curry, black pepper, nutmeg.
Fish odour	Relates to odour of fish (salmon)
Cloying odour	Relates to an unfresh / nauseating odour
Appearance	
Uniformity	Relates to uniformity of the sample, the homogeneity.
Dotted	Relates to the proportion of dots in the sample.
Glossy	Relates to degree of gloss in the surface of the sample
Flavour	
Sour flavour	Relates to a fresh, balanced flavour due to the presence of organic acids.
Sweet taste	Relates to the basic taste sweet (sucrose)
Salt taste	Relates to the basic taste salt (NaCl)
Bitter taste	Relates to the basic taste acid (caffeine)
Umami taste	Relates to the basic taste umami (monosodium glutamate) Relates to flavour of
Metallic flavour	metal (ferro sulphate)
Dairy flavour	Relates to flavour of dairy products like cream and sour cream.
Spice flavour	Relates to flavour of spices like curry, black pepper, nutmeg.
Fish flavour	Relates to flavour of fish (salmon)
Cloying flavour	Relates to an unfresh / nauseating flavour
Aftertaste	Relates to a degree of flavour that remains in the mouth after the sample has been spit out
Texture	
Softness	Mechanical textural attribute relating to the force needed to bite through a sample with molar (1 bite).
Fattiness	Surface textural attribute related to the perception of the amount of fat in a sample.
Granularity	Geometric texture attribute linked to particle size and particle shape in a sample.
Cohesive	Mechanical textural attributes related to the time and number of chews required to make a sample to a state ready for swallowing.
Adhesive	Mechanical textural attribute relating to the force required to remove a substance that sticks to the mouth. Assessed after the sample has been spit out.
Astringent	Describes the complex sensation, accompanied by shrinking, drawing, or puckering of the skin or mucosal surface in the mouth, produced from substances such as tannins.
